# Acidity Functions. Values of the Quantity *p*(*a*_H_*γ*_Cl_) for Buffer Solutions From 0 to 95°C

**DOI:** 10.6028/jres.065A.053

**Published:** 1961-12-01

**Authors:** Roger G. Bates, R. Gary

## Abstract

The thermodynamically defined quantity *p*(*a*_H_*γ*_Cl_) is a useful acidity function for measuring the acidic or basic character of electrolytic solutions. This function can be evaluated from electromotive force data (*E*) for cells without liquid junction consisting of one electrode reversible to hydrogen ions and one electrode reversible to chloride ions. The function is given by

$$p\left(a_{H}\gamma_{\text{Cl}}\right)=\frac{E-E{}^{\circ}}{2.30259RT/F}+\log\;m_{\text{Cl}}.$$

Summarized in 20 tables, the function has been calculated from emf data recorded in the literature for the hydrogen-silver chloride cell. A consistent set of standard potentials (*E*°) and currently accepted values of the natural constants were used. The tables cover a range of *p*(*a*_H_*γ*_Cl_) from 1.6 to 12.5 and a range of temperature from 0 to 95° C. Included are buffer solutions of three different charge types. Ionic strengths vary in the range 0.01 to 0.20. Properties and uses of the acidity function are discussed.

## 1. Introduction

A measure of the acidity or basicity of a solution, applicable in a general way to solutions in all solvent media, has long been sought. Some years ago, Brønsted [1]^1^ suggested that the so-called “proton activity” might constitute a measure of the acidity of one solution relative to another. The concept of acidity is then directly related to the availability of protons for reaction with any base that may be introduced into the solution. It should be emphasized, however, that a high acidity does not indicate an appreciable concentration of free protons, which because of their extremely small size possess intense electrostatic fields and, consequently, can rarely exist uncombined in any liquid medium [2]. The concept of high proton activity therefore implies a relatively loose binding of protons to the basic species present in the solution. In such a solution, the potential of the hydrogen electrode is shifted toward positive values, and the *acidity potential* or *proton energy level* is said to be high [3].

The Brønsted concept of generalized acidity has proved pedagogically important and has facilitated greatly the visualization and interpretation of acid-base reactions where a proton-transfer mechanism is involved. Unfortunately, it is impossible, however, to compare experimentally the proton activities or energy levels in two different media [4]. All attempts to do so are doomed to failure because of the indeterminate character of the liquid-junction potential.

When the composition of the solvent itself is held constant, however, it appears that the acidity or basicity of the solution can be varied over rather wide ranges without changing the liquid-junction potentials appreciably. Consequently, it is often possible to obtain a useful measure of the *relative* acidity of different solutions, provided only that the composition of the solvent is unchanged. Thus a useful experimental *p*H scale exists for aqueous media, and other similar scales could be established for a number of partially aqueous or water-like solvents [4a]. Nevertheless, the acidity numbers would have no exact meaning when compared with those in another solvent or with the water scale.

Although the proton activity cannot be evaluated thermodynamically, the *p*H value of aqueous solutions derives some fundamental meaning from the arbitrary convention on which the numerical values of *p*H are based [5]. If one wishes to estimate thermodynamic equilibrium data through measurements of acidity, the individual *activity* of the proton or hydrogen ion would be much less useful than the hydrogen ion *concentration* or certain other related functions [6]. The simplest “acidity function” is therefore probably *m*_H_, the hydrogen ion concentration, or its negative logarithm, which has been termed *pc*H [7].

The term “acidity function” as used here will signify a useful experimental or operational quantity with exact thermodynamic meaning, the value of which is a function of the acidity of the medium. Acidity functions are usually defined, as is the *p*H value, as the negative logarithm of the concentration of hydrogen ions (protons) multiplied by an appropriate combination of activity coefficients. A given acidity function customarily relates to a particular experimental arrangement or method, perhaps the electromotive force of a particular cell or the spectral absorption of a selected series of suitable indicators. Unlike the *p*H value, these acidity functions derive their usefulness from the unambiguous role that they play in specified chemical equilibria. A good example is provided by the Hammett acidity functions H_0_ and H_−_, which owe their validity to the regularities displayed in the behavior of certain indicators in strongly acid media and in certain other solutions [8, 9].

In 1930, Guggenheim [6] pointed out that an experimental measure of the quantity −log(*m*_H_*γ*_±_) or −log(*m*_H_*γ*_H_*γ*_X_) where X is a univalent anion, would be more useful than −log(*m*_H_*γ*_H_), even if the latter could be determined exactly. In 1936, Hitchcock [10] suggested the use of −log(*m*_H_*γ*_H_*γ*_Cl_), that is, −log(*a*_H_*γ*_Cl_), as a useful acidity function of this type, pointing out that cells with hydrogen and silver-silver chloride electrodes (without a liquid junction) were capable of furnishing very precise values of this quantity in aqueous solutions over a wide range of compositions. This quantity was later termed *pw*H by one of the present authors [7]. Measurements of this function have formed the basis for the establishment of the NBS primary standards of *p*H and for the calculation of the dissociation constants of a number of weak acids and bases in this laboratory. It is likewise implicit in a method suggested by Hamer [11, 12] for the experimental measurement of hydrogen ion concentration or activity.

In 1954, Bates and Schwarzenbach [13] showed how the acidity function *pw*H could be used in combination with spectrophotometric measurements to facilitate greatly the determination of accurate values of the dissociation constants of many uncharged acids with appropriate spectral behavior. This method has been applied successfully to the determination of dissociation constants in aqueous solutions [14] and recently in ethanol-water mixtures [15].

It is the purpose of this paper to summarize in convenient form the most useful data available for the acidity function −log(*m*_H_*γ*_H_*γ*_Cl_). In accordance with a recent suggestion [16], this acidity function is now termed *p*(*a*_H_*γ*_Cl_) instead of *pw*H:

$$p(a_{H}\gamma_{\text{Cl}})\equiv -\log(m_{H}\gamma_{H}\gamma_{\text{Cl}}).$$ (1)

The acidity functions have been calculated from emf measurements for various buffer solutions over a range of temperature, as recorded in the literature. The tabulated values of *p*(*a*_H_*γ*_Cl_) cover the range 1.64 to 12.5 at 25 °C. The range of temperatures extends from 0 to 50 °C, in some instances to 60 or 95 °C.

The original emf data were first converted, where necessary, to absolute volts. The acidity function was then calculated with the use of the most recent values of the standard potential of the cell and with a single, consistent set of the natural constants. In many instances, the functions have been plotted on a large scale and the acidity function interpolated at even values of the ionic strength.

## 2. Procedures

The acidity function *p*(*a*_H_*γ*_Cl_) was calculated from the electromotive force of cells of the type

Pt;H_2_ (g, 1 atm), buffer solution containing soluble chloride, AgCl;Ag.

The calculation was made by the equation

$$p(a_{H}\gamma_{\text{Cl}})=\frac{E-E{}^{\circ}}{2.30259RT/F}+\log\;m_{\text{Cl}}.$$ (2)

In eq (2), *E* is the electromotive force of the above cell, *E*° is the standard potential of this cell [17], *R* is the gas constant (8.31467 j deg^−1^ mole^−1^ [18]), *T* is the absolute temperature in degrees Kelvin *(t* °C + 273.150), and *F* is the faraday (96,495.4 coulombs equiv.^−1^ [18]). The values of *E*° and 2.30259*RT*/*F* (in abs v) used in deriving the acidity functions given in the tables below are summarized in table 1.^2^ Electromotive force data published prior to January 1, 1948, were customarily given in international volts; these values have been converted to absolute volts by multiplying them by the factor 1.00033 [19].

In the use of *p*(*a*_H_*γ*_Cl_) for the determination of thermodynamic data, it is usually necessary to know the ionic strength (*I*) of the buffer solution used and to vary the ionic strength in such a way that an extrapolation to *I*=0 is possible. For this reason, values of *p*(*a*_H_*γ*_Cl_) over a range of ionic strength are needed. For a description of the method of calculating the ionic strength of each buffer system, the reader is referred to the original papers.

When *p*(*a*_H_*γ*_Cl_) was less than 2 and greater than 12, the ionic strength was different at each temperature because of the variation of *m*_H_ (or *m*_OH_). In these cases, solutions of constant stoichiometric concentration were chosen, and the ionic strength at each temperature has been indicated.

In selected instances, it has been possible to give values of the acidity function for chloride-free buffer solutions. These values have been obtained by extrapolation (to the limit of zero concentration of chloride) of data for buffer solutions containing three or more small concentrations of soluble chloride.

## 3. Results

Values of *p*(*a*_H_*γ*_Cl_) over a range of ionic strength and temperature are given in tables 2 to 20 of the appendix (section 6). In general, the maximum uncertainty in *p*(*a*_H_*γ*_Cl_) may be taken to be ±0.004 at 0 to 60 °C. Above 60 °C, the values are probably accurate to ±0.01 unit.

In those cases where the original data were plotted to yield values of *p*(*a*_H_*γ*_Cl_) at round values of ionic strength, or at nearly equally spaced intervals along the ionic strength axis, some smoothing of the original data has naturally occurred. However, no attempt was made to smooth the variation of *p*(*a*_H_*γ*_Cl_) with temperature, and some minor nonuniformities which have their source in the original experimental data may be noted. If it is necessary to obtain *p*(*a*_H_*γ*_Cl_) at temperatures other than those given (or if a smoothed set of data over a range of temperature is desired) the user will probably wish to smooth the data in some way.

Many of the tables show values of −log *K* over a range of temperature. These values have been corrected, where necessary, for changes in *E°* and the natural constants. In the case of formic acid and acetic acid, values of −log *K* are as given in the original papers.

Details concerning the tabulated data for each buffer system are given below. The charge type of the principal acid-base equilibrium responsible for the buffering effect is designated by the charge borne by the base. A buffer composed of HA and A^−^, for example,^3^ is of the charge type −1, whereas buffers of the type HA^−^, A^=^ and HB^+^, B are of charge type −2 and 0, respectively.

KH_3_(C_2_O_4_)_2_ (*m*) [20] (table 2). Buffer system: H_2_C_2_O_4_, ${\text{HC}}_{2}O_{4}^{-}$. Charge type: −1. Ionic strength: *m*+*m*_H_.
KH_2_PO_4_(*m*_1_), HCl (*m*_2_) [21] (table 3). Buffer system: H_3_PO_4_, $H_{2}{\text{PO}}_{4}^{-}$. Charge type: −1. Ionic strength: *m*_1_+*m*_H_.
HCOOH (*m*_1_), HCOOK (*m*_2_), KCl (*m*_3_) [22] (table 4). Buffer system: HCOOH, HCOO^−^. Charge type: −1. Ionic strength: *m*_2_+*m*_3_+*m*_H_.
NaH succinate (*m*_1_), HCl (*m*_2_) [23] (table 5). Buffer system: H_2_Suc, HSuc^−^ (where Suc=succinate). Charge type: −1. Ionic strength: *m*_1_+*m*_H_+*m*_Suc_^=^.
KH phthalate (0.05*m*) [24] (table 6). Buffer systems: H_2_Ph,HPh^−^ and HPh^−^, Ph^=^ (where Ph= phthalate). Charge types: −1 and −2. Ionic strength: 0.0533.
CH_3_COOH (*m*_1_), CH_3_COONa (*m*_2_), NaCl (*m*_3_) [25] (table 7a). Buffer system: CH_3_COOH, CH_3_COO^−^. Charge type: −1. Ionic strength: *m*_2_+*m*_3_+*m*_H_.
CH_3_COOH (*m*), CH_3_COONa (*m*), NaCl (*m*) [26] (table 7b). Buffer system: CH_3_COOH, CH_3_COO^−^. Charge type: −1. Ionic strength: 2*m*+*m*_H_.
NaH succinate (*m*), NaCl (*m*) [27] (table 8). Buffer systems: H_2_Suc, HSuc^−^ and HSuc^−^, Suc^=^, (where Suc=succinate). Charge types: −1 and −2. Ionic strength: $2m+2m_{H}+m_{H_{2}\text{Suc}}$.
KH phthalate (*m*_1_), K_2_ phthalate (*m*_2_), KCl (*m*_3_) [28] (table 9). Buffer systems: HPh^−^, Ph^=^ (where Ph=phthalate). Charge type: −2. Ionic strength: *m*_1_+3*m*_2_+*m*_3_+2*m*_H_.
NaH succinate (*m*), Na_2_ succinate (*m*) [29] (table 10). Buffer system: HSuc− Suc^=^ (where Suc=succinate). Charge type: −2. Ionic strength: $4m+2m_{H}+m_{H_{2}\text{Suc}}$.
KH_2_PO_4_ (*m*), Na_2_HPO_4_ (*m*) [30] (table 11). Buffer system: $H_{2}{\text{PO}}_{4}^{-}$, ${\text{HPO}}_{4}^{-}$. Charge type: −2. Ionic strength: 4*m*.
KH *p*-phenolsulfonate (*m*_1_), KNa *p*-phenolatesulfonate (*m*_2_), NaCl (*m*_3_) [31] (table 12). Buffer system: HPs^−^, Ps^=^ (where Ps=*p*^_^phenolatesulfonate). Charge type: −2. Ionic strength: *m*_1_+3*m*_2_+*m*_3_−*m*_OH_.
Na_2_B_4_O_7_ (*m*_1_), NaCl (*m*_2_) [32] (table 13). Buffer system: H_3_BO_3_, ${\text{BO}}_{2}^{-}$. Charge type: −1. Ionic strength : 2*m*_1_+*m*_2_−*m*_OH_.
Triethanolamine (*m*_1_), HCl (*m*_2_) [33] (table 14). Buffer system: (HOC_2_H_4_)_3_NH^+^, (HOC_2_H_4_)_3_N. Charge type: 0. Ionic strength: *m*_2_.
Tris(hydroxymethyl)aminomethane (*m*_1_), HCl (*m*_2_) [34] (table 15). Buffer system: (HOCH_2_)_3_CNH_3_^+^, (HOCH_2_)_3_CNH_2_. Charge type: 0. Ionic strength: *m*_2_.
4-Aminopyridine (*m*_1_), HCl (*m*_2_) [35] (table 16). Buffer system: H_2_NC_5_H_4_NH^+^, H_2_NC_5_H_4_N. Charge type: 0. Ionic strength: *m*_2_.
Monoetlianolamine (*m*_1_), HCl (*m*_2_) [36] (table 17). Buffer system: HOC_2_H_4_NH_3_^+^, HOC_2_H_4_NH_2_. Charge type: 0. Ionic strength: *m*_2_+*m*_OH_.
Piperidine (*m*_1_), HCl (*m*_2_) [37] (table 18). Buffer system: C_5_H_10_NH_2_^+^, C_5_H_10_NH. Charge type: 0. Ionic strength: *m*_2_+*m*_OH_.
Calcium hydroxide (*m*) [38] (table 19). Buffer system: CaOH^+^, Ca^++^. Ionic strength: 2*m*_OH_−*m*.

*p*(*a*_H_*γ*_Cl_) in some solutions at 60 to 95° C [39] (table 20). Charge types: −1 (tetroxalate); −1, −2 (tartrate); −1, −2 (phthalate); −2 (phosphate); −1 (borax). Ionic strength: 0.073 (tetroxalate); 0.04 (tartrate); 0.053 (phthalate); 0.1 (phosphate); 0.02 (borax).

## 4. Uses of *p*(*a*_H_*γ*_Cl_)

### 4.1. Hydrogen Ion Concentrations

From the definition of the acidity function *p*(*a*_H_*γ*_Cl_) given in eq (1), it is evident that the hydrogen ion concentration (*m*_H_) is formally related to the acidity function as follows:

$$-\log\;m_{H}=p(a_{H}\gamma_{\text{Cl}})+\log\;(\gamma_{H}\gamma_{\text{Cl}})=p(a_{H}\gamma_{\text{Cl}})+2\;\log\;\gamma_{\pm }.$$ (3)

In this equation, *γ_±_* is the mean ionic activity coefficient of hydrochloric acid in the buffer solution for which the acidity function has been obtained.

Although each of the terms in eq (3) is thermodynamically defined, the mean activity coefficients of hydrochloric acid in buffer solutions have not often been determined. Largely as a result of the fact that the concentration of hydrogen ion in buffer solutions is rarely known, the determinations of these mean activity coefficients is not usually a simple matter. It is possible, nonetheless, to derive approximate hydrogen ion concentrations from the acidity function when the ionic strength does not exceed 0.1. Perhaps the best procedure is to utilize the Davies equation [40], as modified by Robinson [41], to compute *γ*_±_:

$$-\log\;\gamma_{\pm }=\frac{A\sqrt{I}}{1+\sqrt{I}}-0.2\;I,$$ (4)

where *A*, the Debye-Hückel slope, is a function of temperature. Values of *A* have been tabulated by Robinson and Stokes [42]. This equation reproduces rather satisfactorily the known mean activity coefficients of hydrochloric acid in the pure aqueous solution or in the presence of alkali halides in the low range of ionic strengths.

### 4.2. Hydroxide Ion Concentrations

A useful application of *p*(*a*_H_*γ*_Cl_) has been suggested by Bates, Siegel, and Acree [43]. According to this proposal, hydroxide ion concentrations in buffer solutions can be calculated by combining the acidity function with the ion-product constant, *K_w_*, for water, values of which are listed by Harned and Owen [44]. Thus,

$$-\log\;K_{w}-p(a_{H}\gamma_{\text{Cl}})=-\log\;m_{\text{OH}}+\log\;a_{H_{2}O}\gamma_{\text{Cl}}/\gamma_{\text{OH}}\approx -\log\;m_{\text{OH}}.$$ (5)

The activity of water, $a_{H_{2}O}$, does not depart greatly from unity in dilute solutions; furthermore, both the hydroxide and chloride ions bear the same charge, and the ratio of their activity coefficients is likewise nearly unity when the ionic strength is less than 0.1. Electromotive force data in the literature show that the second term on the right of eq (5) amounts to only 0.013 in a mixture of potassium hydroxide (0.01 *m*) and potassium chloride (0.1 *m*) [45], and to only 0.003 in a mixture of sodium hydroxide (0.01 *m*) and sodium chloride (0.1 *m*) [46]. The ionic strength of both of these solutions is 0.11. It follows, therefore, that the left side of eq (5) is very often a useful approximation to −log *m*_OH_.

### 4.3. Activity Coefficients

By combining acidity potentials with the dissociation constants for the buffer acids or bases, one can derive certain useful thermodynamic combinations of activity coefficients for the respective buffer systems. If HB and B represent, respectively, the weak acid and its conjugate base, which together are responsible for the buffering effect, one obtains

$$-\log\;\frac{\gamma_{\text{Cl}}\gamma_{\text{HB}}}{\gamma_{B}}=p(a_{H}\gamma_{\text{Cl}})+\log\;K+\log\;\frac{m_{\text{HB}}}{m_{B}},$$ (6)

where *K* is the dissociation constant of the buffer acid given in the appended tables. For the various buffers listed in the tables, the buffer pair HB, B may have the following charges: HB^+^, B; HB, B^−^; or HB^−^, B^=^.

In order to utilize eq (6) for the calculation of activity coefficients, it is evidently necessary to know the ratio of the concentrations of the buffer species in the solution. When the value of *p*(*a*_H_*γ*_Cl_) lies between 4 and 10, it is usually possible to use directly the stoichiometric ratio of molalities given in the tables. For dilute solutions of low and high acidity, however, the hydrolysis of HB or B may be sufficiently extensive that the stoichiometric ratio must be corrected accordingly. This is easily accomplished with the aid of hydrogen ion concentrations or hydroxyl ion concentrations estimated as indicated in the above paragraphs. Thus,

$$\frac{m_{\text{HB}}}{m_{B}}=\frac{m_{\text{HB}}^{\circ }-m_{H}+m_{\text{OH}}}{m_{B}^{\circ }+m_{H}-m_{\text{OH}}}$$ (7)

where $m_{\text{HB}}^{{}^{\circ}}$ and $m_{B}^{{}^{\circ}}$ represent the stoichiometric molalities of the two species.

### 4.4. *pa*_H_ Values

The acidity function *p*(*a*_H_*γ*_Cl_) can be used to estimate−log *a*_H_ when values of this quantity are needed. Indeed, the standard reference values for the buffer solutions used by the National Bureau of Standards to define the *p*H scale are derived in this way. They are designated *p*H_s_ or *pa*_H_ and are computed by the following equation:

$$pH_{s}=p(a_{H}\gamma_{\text{Cl}})+\log\;\gamma_{\text{Cl}}$$ (8)

$$=p(a_{H}\gamma_{\text{Cl}})-\frac{A\surd I}{1+1.5\sqrt{I}},$$ (8a)

where *A* is again the Debye-Hückel slope. The activity coefficient of a single ionic species such as chloride is, of course, not thermodynamically defined. The convention used to evaluate this coefficient for the determination of standard *p*H values by eq (8a) is that recently proposed to International Union of Pure and Applied Chemistry by Bates and Guggenheim [16].

### 4.5. Dissociation Constants

Perhaps the most fruitful use of the acidity function *p*(*a*_H_*γ*_Cl_) is in the determination of the dissociation constants of uncharged acids by means of spectrophotometric measurements, when the spectral absorptions of the conjugate acid and base species differ sufficiently to permit the ratio of the concentrations of these forms in the solution to be established accurately by absorption spectrophotometry [13]. The acid-base system will be written HIn,In^−^ to designate its indicator properties. The acid HIn and its conjugate base In^−^ are added to suitable “regulating” buffer solutions (HB,B) in quantities so small that the acidity function of the buffer is unaffected by their presence. The buffers selected must (a) have acidity functions that differ no more than about 1 unit from −log *K*_HIn_ for best results, and (b) must not absorb light in the wavelength region where the useful absorption bands of In^−^ or HIn are located. The dissociation constant is then calculated by the expression

$$-\log\;K_{\text{HIn}}=p(a_{H}\gamma_{\text{Cl}})-\log\;\frac{m_{{\text{In}}^{-}}}{m_{\text{HIn}}}+\log\;\frac{\gamma_{\text{HIn}}\gamma_{{\text{Cl}}^{-}}}{\gamma_{{\text{In}}^{-}}}.$$ (9)

It is well known that quantities such as the last term of eq (9) are not only small in magnitude but vary linearly with ionic strength in dilute solutions. For this reason, two or more buffer solutions of different ionic strengths are chosen, and the apparent values of −log *K*_HIn_ are plotted as a function of ionic strength and extrapolated to infinite dilution. This method has been applied with success to the determination of dissociation constants of substituted phenols and substituted anilines in aqueous solution [13, 14] and to the determination of the dissociation constant of anisic acid in ethanol-water mixtures [15]. For the most refined measurements, corrections should be applied for the effect of the system HIn, In^−^ upon the acidity function, as Robinson and Biggs have shown [41].

From an examination of the last term in eq (9), it is evident that the acidity function *p*(*a*_H_*γ*_Cl_) is more suitable for determining the dissociation constants of uncharged indicator acids, HIn, than for those of acids of other charge types, because of the near equality of the activity coefficients of ions of like charge in the same solution. For indicator acids of other charge types, Bates and Schwarzenbach [13] suggested that the control buffer solution (HB,B) selected be of the same charge type as the indicator acid system (HIn,In). If, then, the equations are written in terms of the dissociation constant of the buffer acid HB (whatever its charge type) instead of the acidity function, the same advantage of a cancellation of the activity coefficients can be gained. Regardless of charge type, the applicable equation is

$$-\log\;K_{\text{HIn}}=-\log\;K_{\text{HB}}+\log\;\frac{m_{\text{HIn}}}{m_{\text{In}}}-\log\frac{m_{\text{HB}}}{m_{B}}+\log\;\frac{\gamma_{\text{HIn}}\gamma_{B}}{\gamma_{\text{In}}\gamma_{\text{HB}}}.$$ (10)

The last term is again small and varies linearly with ionic strength. The ratio of concentrations of HIn and In is determined spectrophotometrically, and that of HB and B is derived from the composition of the control buffer solution. Values of −log *K*_HB_ are listed in the tables. These have been corrected, where necessary, to conform to the values of *E°* and 2.30259*RT*/*F* given in table 1.

### 4.6. Nonaqueous and Partially Aqueous Media

The Hammett acidity functions H_0_ and H_−_ have been conspicuously successful in correlating proton levels with the extent of acid-base reactions and with reaction rates, particularly in concentrated aqueous solutions of the strong acids [8, 9]. These acidity functions are defined in terms of the spectral absorption of a series of selected indicators. They depend for their validity on the regularities shown by the ratio of activity coefficients, *γ*_HIn_/*γ*_In_, of these indicator forms in strongly acidic media. Unfortunately, however, the values of *γ*_HIn_/*γ*_In_ for different indicator acids of a given charge type cannot be expected to be equal in the same medium any more than are the values of *γ*_HA_/*γ*_A_ for other acids [47, 48]. In many media, indeed, indicator behavior displays even less regularity than it does in solutions of strong acids, it appears. Without specifying the charge type of the indicator, one may define the Hammett function as follows:

$$H=-\log\;a_{H}\frac{\gamma_{\text{In}}}{\gamma_{\text{HIn}}}.$$ (11)

The subscripts in the symbols H_0_ and H_−_ signify that the base form of the indicator (In) is, respectively, either an uncharged molecule or a singly-charged anion.

It is apparent that the acidity function *p*(*a*_H_*γ*_Cl_) has very much the same form as the Hammett function H_−_. It seems possible that *p*(*a*_H_*γ*_Cl_) may find use as a practical index of the acidity of a wide variety of media to which it has not as yet been applied. The Hammett functions derive their usefulness in part from the fact that the aqueous standard state is maintained throughout. The function therefore reflects, to a degree, the changes of acidity from one medium to another. To preserve this same utility, values of *p*(*a*_H_*γ*_Cl_) should likewise be based upon standard potentials for the hydrogen-silver chloride cell in water, no matter what the composition of the medium in which the measurement of electromotive force is made.

Even so, the changes in *p*(*a*_H_*γ*_Cl_) cannot be expected to parallel the changes in H_−_. In the first place, it must be recognized that profound changes in the character of the solvent medium will affect *γ*_Cl−_ and *γ*_In−_ in different ways, depending upon the susceptibilities of these two ions to solvation in the medium concerned and the abilities of the ions to “sort out’’ the molecules of mixed solvents. Furthermore, *γ*_HIn_, which occurs in the expression for H_−_, is not unaffected by changes of solvent, even though the species HIn bears no charge. According to Kolthoff, Lingane, and Larson [49], the activity coefficients of uncharged buffer acids decrease from values near unity to values in the vicinity of 0.01 when the solvent composition passes from pure water to pure ethanol or pure methanol.

In addition, determinations of *p*(*a*_H_*γ*_Cl_) are, of course, limited to media in which the hydrogen electrode and the silver-silver chloride electrode give reproducible potentials. It is necessary, therefore, to add a small known concentration of soluble chloride to the medium whose acidity potential is to be determined. It is probably true, however, that the difficulties in determining *p*(*a*_H_*γ*_Cl_) are no more restrictive than those affecting the determination of the Hammett functions. The use of the thermodynamically defined quantity *p*(*a*_H_*γ*_Cl_) in a variety of media as an index of the proton availability (still experimentally inaccessible) therefore merits further study.

## Figures and Tables

**Table 1:** Summary of the values of E° and 2.30259RT/F used to compute the acidity function p(*a*_*H*_γ_*Cl*_)

*t*	*E°*	*2.30259RT*/*F*
		
*°C*	*abs v*	*abs v*
0	0.23655	0.054195
5	.23413	.055187
10	.23142	.056183
15	.22857	.057171
20	.22557	.058163
25	.22234	.059155
30	.21904	.060147
35	.21565	.061139
38	.21352	.061734
40	.21208	.062131
45	.20835	.063123
50	.20449	.064115
55	.20056	.065107
60	.19649	.066099
70	.18782	.068083
80	.17873	.070067
90	.16952	.072051
95	.16511	.073043

## Footnotes

1 Figures in brackets indicate the literature references at the end of this paper.

2 The planned change to the C–12 scale of atomic weights will affect both *R* and *F* in equal proportion and, hence, will not change the values of 2.30259*RT/F.*

3 In these examples. A^–^, A^=^, and B are all bases in the Brønsted sense, in that they are able to accept protons.

## 6. Appendix. Tables of Data

**Table 2:** p(*a*_*H*_γ_*Cl*_) in solutions of potassium textroxalate (*m*)

*t*	*m*=0.01000		0.02500		0.05000		0.10000	
			
	*I*	*p*(*a*_H_*γ*_Cl_)	*I*	*p*(*a*_H_*γ*_Cl_)	*I*	*p*(*a*_H_*γ*_Cl_)	*I*	*p*(*a*_H_*γ*_Cl_)
								
*°C*								
0	0.0181	2.206	0. 0417	1.932	0.0772	1.765	……….	……….
5	.0181	2.202	.0416	1.934	.0770	1.764	……….	……….
10	.0181	2.207	.0416	1.938	.0767	1.765	……….	……….
15	.0181	2.210	.0415	1.940	.0765	1.769	0.1409	1.623
20	.0180	2.212	.0414	1.942	.0763	1.773	.1404	1.627
25	.0180	2.214	.0413	1.947	.0760	1.780	.1400	1.640
30	.0180	2.218	.0412	1.952	.0758	1.785	.1396	1.643
35	.0179	2.221	.0410	1.957	.0755	1.792	.1394	1.651
40	.0179	2.220	.0408	1.962	.0753	1.797	.1391	1.660
45	.0178	2.230	.0407	1.968	.0751	1.803	.1389	1.670
50	.0177	2.234	.0405	1.970	.0749	1.811	.1387	1.681
55	.0176	2.238	.0403	1.981	.0747	1.819	.1385	1.692
60	.0175	2.239	.0401	1.987	.0744	1.824	.1383	1.702

**Table 3:** (p*a*_*H*_γ_*Cl*_) in solutions containing potassium dihydrogen phosphate (*m*_1_) and hydrochloric acid (*m*_2_); *m*_2_=0.8055*m*_1_

*t*	−log *K*_1_	*m*_1_=0.02600		0.03010		0.03841		0.04684		0.05533		0.06386		0.07249		0.08124	
							
		*I*	*p*(*a*_H_*γ*_Cl_)	*I*	*p*(*a*_H_*γ*_Cl_)	*I*	*p*(*a*_H_*γ*_Cl_)	*I*	*p*(*a*_H_*γ*_Cl_)	*I*	*p*(*a*_H_*γ*_Cl_)	*I*	*p*(*a*_H_*γ*_Cl_)	*I*	*p*(*a*_H_*γ*_Cl_)	*I*	*p*(*a*_H_*γ*_Cl_)
																	
*°C*																	
0	2.056	0.0359	2.156	0.0410	2.120	0.0512	2.065	0.0614	2.022	0.0716	1.986	0.0818	1.954	0.0920	1.928	0.1023	1.904
5	2.070	.0357	2.164	.0408	2.129	.0510	2.074	.0611	2.030	.0713	1.994	.0815	1.963	.0916	1.937	.1018	1.913
10	2.088	.0355	2.172	.0406	2.137	.0507	2.083	.0609	2.040	.0710	2.003	.0811	1.973	.0912	1.947	.1013	1.923
15	2.106	.0353	2.180	.0404	2.146	.0506	2.092	.0606	2.049	.0707	2.013	.0807	1.983	.0908	1.957	.1009	1.934
20	2.127	.0352	2.189	.0402	2.156	.0503	2.102	.0603	2.059	.0703	2.024	.0804	1.994	.0904	1.968	.1004	1.945
25	2.150	.0350	2.201	.0400	2.166	.0500	2.115	.0600	2.071	.0700	2.036	.0800	2.006	.0900	1.982	.1000	1.958
30	2.169	.0348	2.212	.0398	2.178	.0497	2.127	.9597	2.083	.0696	2.049	.0796	2.019	.0895	1.995	.0995	1.971
35	2.195	.0346	2.223	.0395	2.190	.0494	2.139	.0593	2.097	.0692	2.062	.0791	2.032	.0890	2.008	.0989	1.985
40	2.223	.0344	2.234	.0393	2.202	.0491	2.151	.0590	2.109	.0688	2.074	.0787	2.045	.0885	2.020	.0984	1.998
45	2.249	.0341	2.246	.0390	2.215	.0488	2.164	.0586	2.122	.0685	2.088	.0782	2.058	.0881	2.034	.0979	2.012
50	2.276	.0339	2.258	.0388	2.228	.0485	2.177	.0583	2.136	.0681	2.102	.0778	2.073	.0876	2.049	.0973	2.028
55	2.305	.0337	2.271	.0385	2.241	.0482	2.191	.0580	2.150	.0677	2.117	.0774	2.089	.0871	2.065	.0968	2.046
60	2.335	.0335	2.285	.0383	2.255	.0479	2.205	.0576	2.165	.0673	2.133	.0769	2.106	.0866	2.082	.0963	2.064

**Table 4:** p(*a*_*H*_γ_*Cl*_) in solutions containing formic acid (*m*_1_), potassium formate (*m*_2_), and potassium chloride (*m*_3_); *m*_2_=1.2467 *m*_1_; *m*_3_=1.0789 *m*_1_

*t*	−log *K*	*I*=0.02	0.03	0.04	0.05	0.06	0.07	0.08	0.10	0.12	0.15
									
		*m*_1_=0.008544	0.012840	0.017136	0.02143	0.02573	0.03004	0.03434	0.04294	0.05154	0.06444
											
*°C*											
0	3.786	3.894	3.892	3.891	3.891	3.891	3.891	3.892	3.893	3.895	3.898
5	3.772	3.882	3.879	3.878	3.878	3.878	3.878	3.879	3.880	3.881	3.883
10	3.762	3.875	3.871	3.870	3.869	3.869	3.870	3.870	3.872	3.873	3.875
15	3.757	3.870	3.867	3.865	3.864	3.864	3.864	3.865	3.866	3.867	3.869
20	3.753	3.866	3.864	3.862	3.862	3.862	3.862	3.862	3.863	3.864	3.866
25	3.752	3.867	3.864	3.862	3.861	3.861	3.862	3.862	3.863	3.864	3.866
30	3.752	3.868	3.865	3.863	3.862	3.862	3.863	3.863	3.865	3.866	3.868
35	3.758	3.871	3 868	3.867	3.866	3.866	3.866	3.866	3.867	3.868	3.870
40	3.766	3.886	3.875	3.872	3.872	3.871	3.871	3.872	3.872	3.873	3.875
45	3.773	3.885	3.881	3.880	3.879	3.878	3.878	3.878	3.878	3.879	3.880
50	3.782	3.894	3.890	3.888	3.887	3.887	3.887	3.888	3.888	3.889	3.890
55	3.794	3.904	3.900	3.898	3.897	3.897	3.898	3.898	3.899	3.900	3.901
60	3.809	3.917	3.913	3.911	3.910	3.910	3.910	3.910	3.911	3.912	3.913

**Table 5:** p(*a*_*H*_γ_*Cl*_) in solutions containing sodium hydrogen succinate (*m*_1_) and hydrochloric acid (*m*_2_); *m*_2_=0.6667 *m*_1_

*t*	−log *K*_1_	*I*=0.01516	0.02017	0.02517	0.03018	0.04019	0.05019	0.06020	0.07020	0.08021	0.10021
									
		*m*_1_=0.015000	0.02000	0.02500	0.03000	0.04000	0.05000	0.06000	0.07000	0.08000	0.10000
											
*°C*											
0	4.284	3.970	3.967	3.964	3.962	3.958	3.955	3.953	3.952	3.950	3.948
5	4.261	3.950	3.947	3.945	3.942	3.938	3.935	3.933	3.931	3.929	3.925
10	4.245	3.933	3.930	3.927	3.925	3.921	3.918	3.916	3.914	3.913	3.909
15	4.231	3.920	3.917	3.914	3.912	3.908	3.905	3.902	3.900	3.898	3.895
20	4.218	3.909	3.905	3.902	3.899	3.895	3.893	3.890	3.888	3.886	3.883
25	4.209	3.902	3.898	3.895	3.892	3.887	3.884	3.882	3.880	3.878	3.875
30	4.200	3.894	3.890	3.886	3.884	3.880	3.877	3.874	3.872	3.871	3.867
35	4.192	3.888	3.884	3.881	3.878	3.873	3.870	3.868	3.866	3.864	3.861
40	4.189	3.885	3.881	3.878	3.875	3.870	3.867	3.865	3.863	3.861	3.858
45	4.186	3.884	3.880	3.876	3.873	3.868	3.865	3.862	3.860	3.858	3.855
50	4.185	3.882	3.878	3.874	3.871	3.867	3.864	3.861	3.859	3.857	3.853

**Table 6:** p(*a*_*H*_γ_*Cl*_) in solutions containing 0.05-*m* potassium hydrogen phthalate (*I*=0.0533)

*t*	*p*(*a*_H_*γ*_Cl_)
	
*°C*	
0	4.090
5	4.084
10	4.082
15	4.083
20	4.087
25	4.096
30	4.104
35	4.113
40	4.125
45	4.138
50	4.155

**Table 7a:** p(*a*_*H*_γ_*Cl*_) in solutions containing acetic acid (*m*_1_), sodium acetate (*m*_2_), and sodium chloride (*m*_3_); *m*_2_=0.9624 *m*_1_; *m*_3_=1.0243 *m*_1_

*t*	−log *K*	*I*=0.01	0.02	0.03	0.04	0.05	0.06	0.07	0.08	0.09	0.10
									
		*m*_1_=0.005034	0.010067	0.015100	0.02013	0.02517	0.03020	0.03523	0.04027	0.04530	0.05034
											
*°C*											
0	4.781	4.768	4.769	4.770	4.771	4.772	4.773	4.773	4.774	4.774	4.775
5	4.770	4.757	4.758	4.758	4.759	4.759	4.760	4.761	4.761	4.762	4.762
10	4.762	4.750	4.751	4.752	4.752	4.753	4.753	4.754	4.754	4.755	4.756
15	4.758	4.746	4.747	4.747	4.748	4.748	4.748	4.749	4.749	4.750	4.750
20	4.756	4.746	4.747	4.747	4.747	4.747	4.747	4.747	4.747	4.748	4.748
25	4.756	4.746	4.747	4.747	4.747	4.747	4.747	4.748	4.748	4.748	4.748
30	4.757	4.748	4.748	4.748	4.748	4.749	4.749	4.749	4.749	4.749	4.750
35	4.762	4.752	4.752	4.752	4.752	4.752	4.752	4.752	4.752	4.752	4.752

**Table 7b:** p(*a*_*H*_γ_*Cl*_) in solutions containing acetic acid (*m*), sodium acetate (*m*), and sodium chloride (*m*)

*t*	−log *K*	*I*=0.01	0.02	0.03	0.04	0.05	0.06	0.07	0.08	0.09	0.10
									
		*m*=0.005000	0.01000	0.01500	0.02000	0.02500	0.03000	0.03500	0.04000	0.04500	0.05000
											
*°C*											
35	4.762	4.768	4.768	4.769	4.770	4.770	4.770	4.770	4.770	4.770	4.770
40	4.769	4.775	4.775	4.775	4.775	4.775	4.775	4.775	4.775	4.775	4.775
45	4.777	4.781	4.782	4.782	4.782	4.782	4.782	4.782	4.783	4.783	4.783
50	4.787	4.791	4.790	4.790	4.790	4.790	4.790	4.790	4.790	4.790	4.790
55	4.799	4.801	4.801	4.800	4.800	4.800	4.800	4.800	4.800	4.800	4.800
60	4.812	4.813	4.813	4.812	4.812	4.812	4.812	4.812	4.811	4.811	4.811

**Table 8:** p(*a*_*H*_γ_*Cl*_) in solutions containing sodium acid succinate (*m*) and sodium chloride (*m*)

*t*	*I*=0.0418	0.0681	0.108	0.158	0.217
				
	*m*=0.019390	0.03155	0.05012	0.07296	0.10000
					
*°C*					
0	4.915	4.901	4.887	4.877	4.867
5	4.894	4.879	4.866	4.855	4.845
10	4.880	4.864	4.851	4.839	4.829
15	4.868	4.853	4.838	4.827	4.817
20	4.861	4.847	4.831	4.817	4.809
25	4.853	4.838	4.826	4.814	4.802
30	4.852	4.839	4.822	4.809	4.798
35	4.850	4.837	4.820	4.805	4.796
40	4.851	4.838	4.821	4.806	4.796
45	4.855	4.842	4.823	4.810	4.799
50	4.860	4.848	4.827	4.815	4.804

**Table 9:** −p(*a*_*H*_γ_*Cl*_) in solutions containing potassium hydrogen phthalate (*m*_1_), dipotassium phthalate (*m*_2_), and potassium chloride (*m*_3_); *m*_2_=1.0057 *m*_1_ and *m*_3_=1.0014 *m*_1_

*t*	−log *K_2_*	I=0.01	0.02	0.03	0.04	0.05	0.06	0.07	0.08	0.09	0.10
									
		*m*_1_=0.002007	0.004015	0.006022	0.008031	0.010037	0.012044	0.014052	0.016059	0.018067	0.022007
											
*°C*											
0	5.432	5.349	5.316	5.295	5.279	5.265	5.253	5.242	5.232	5.223	5.216
5	5.417	5.331	5.300	5.278	5.262	5.248	5.236	5.225	5.215	5.206	5.199
10	5.408	5.322	5.292	5.270	5.253	5.240	5.227	5.216	5.206	5.197	5.190
15	5.404	5 317	5.286	5.264	5.246	5.232	5.220	5.209	5.200	5.191	5.183
20	5.404	5.316	5.284	5.262	5.245	5.230	5.218	5.207	5.197	5.188	5.180
25	5.411	5.323	5.290	5.268	5.251	5.237	5.224	5.212	5.202	5.192	5.184
30	5.419	5.329	5.297	5.275	5.257	5.242	5.229	5.218	5.208	5.199	5.190
35	5.428	5.338	5.305	5.281	5.262	5.247	5.235	5.224	5.214	5.205	5.197
40	5.443	5.352	5.319	5.296	5.278	5.263	5.250	5.239	5.228	5.218	5.209
45	5.462	5.368	5.335	5.312	5.293	5.278	5.265	5.253	5.243	5.233	5.225
50	5.484	5.390	5.356	5.333	5.314	5.299	5.285	5.274	5.263	5.253	5.244
55	5.510	5.415	5.382	5.357	5.338	5.322	5.309	5.298	5.287	5.278	5.269
60	5.538	5.444	5.409	5.384	5.365	5.349	5.335	5.324	5.314	5.305	5.296

**Table 10:** −p(*a*_*H*_γ_*Cl*_) in solutions containing sodium hydrogen succinate (*m*) and disodium succinate (*m*)

*t*	−log *K*_2_	I=0.041	0.101	0.202
		
		*m*=0.01000	0.02500	0.05000
				
°*C*				
0	5.675	5.599	5.560	5.531
5	5.660	5.582	5.542	5.513
10	5.649	5.569	5.528	5.498
15	5.642	5.561	5.519	5.488
20	5.638	5.555	5.513	5.481
25	5.638	5.553	5.511	5.477
30	5.641	5.553	5.511	5.476
35	5.647	5.556	5.514	5.477
38	5.652	5.559	5.517	5.479
40	5.656	5.562	5.520	5.481

**Table 11:** −p(*a*_*H*_γ_*Cl*_) in solutions containing potassium dihydrogen phosphate (*m*) and disodium hydrogen phosphate (*m*)

*t*	−log *K*_2_	*I*=0.01	0.02	0.03	0.04	0.05	0.06	0.08	0.10	0.12	0.15	0.20
										
		*m*=0.002500	0.004000	0.007500	0.01000	0.01250	0.01500	0.02000	0.02500	0.03000	0.03750	0.05000
												
*°C*												
0	7.313	7.226	7.196	7.174	7.157	7.143	7.130	7.109	7.091	7.076	7.056	7.029
5	7.280	7.193	7.162	7.141	7.123	7.109	7.096	7.075	7.057	7.042	7.022	6.995
10	7.253	7.165	7.134	7.112	7.095	7.081	7.068	7.047	7.029	7.014	6.994	6.969
15	7.230	7.142	7.111	7.089	7.072	7.057	7.045	7.024	7.006	6.992	6.971	6.945
20	7.213	7.124	7.093	7.072	7.054	7.039	7.027	7.005	6.988	6.973	6.953	6.927
25	7.200	7.111	7.080	7.058	7.040	7.026	7.013	6.992	6.974	6.959	6.940	6.912
30	7.192	7.102	7.070	7.048	7.031	7.016	7.003	6.982	6.964	6.949	6.929	6.902
35	7.186	7.095	7.064	7.041	7.024	7.009	6.996	6.974	6.956	6.941	6.921	6.894
40	7.182	7.090	7.059	7.036	7.019	7.004	6.991	6.969	6.951	6.936	6.917	6.890
45	7.181	7.089	7.057	7.034	7.016	7.001	6.989	6.967	6.949	6.934	6.914	6.886
50	7.182	7.089	7.057	7.034	7.016	7.001	6.988	6.966	6.948	6.933	6.913	6.885
55	7.185	7.091	7.059	7.036	7.018	7.003	6.990	6.968	6.950	6.935	6.915	6.888
60	7.191	7.096	7.064	7.041	7.023	7.008	6.995	6.973	6.954	6.939	6.919	6.892

**Table 12:** p(*a*_*H*_γ_*Cl*_) in solutions containing potassium p-phenolsulfonate (*m*_1_), potassium sodium *p*-phenolatesulfonate (*m*_2_), and sodium chloride (*m*_3_); *m*_2_=1.0120 *m*_1_ and *m*_3_=0.9994 *m*_1_

*t*	−log *K*_2_	*I*=0.02	0.03	0.04	0.05	0.06	0.07	0.08	0.09	0.10
								
		*m*_1_=0.003972	0.005958	0.007943	0.009929	0.011915	0.013901	0.015887	0.017873	0.019858
										
*°C*										
0	9.352	9.258	9.244	9.232	9.223	9.216	9.208	9.203	9.198	9.194
5	9.282	9.189	9.176	9.164	9.154	9.146	9.139	9.134	9.129	9.124
10	9.219	9.124	9.111	9.099	9.090	9.082	9.075	9.069	9.064	9.059
15	9.159	9.063	9.050	9.040	9.030	9.022	9.015	9.009	9.004	8.999
20	9.105	9.007	8.994	8.982	8.972	8.964	8.958	8.952	8.946	8.942
25	9.055	8.957	8.944	8.932	8.922	8.914	8.907	8.901	8.896	8.891
30	9.008	8.908	8.895	8.884	8.874	8.865	8.857	8.851	8.845	8.840
35	8.962	8.861	8.848	8.836	8.827	8.818	8.810	8.804	8.798	8.793
40	8.922	8.818	8.804	8.793	8.784	8.776	8.768	8.761	8.755	8.750
45	8.883	8.778	8.766	8.754	8.744	8.735	8.727	8.721	8.715	8.710
50	8.848	8.740	8.729	8.717	8.707	8.698	8.690	8.684	8.678	8.674
55	8.814	8.702	8.692	8.681	8.671	8.662	8.654	8.648	8.642	8.638
60	8.784	8.669	8.658	8.648	8.638	8.629	8.620	8.614	8.609	8.605

**Table 13:** p(*a*_*H*_γ_*Cl*_) in solutions containing borax (*Na*_2_*B*_4_*O*_7_) (*m*_1_) and sodium chloride (**m**_2_); *m*_2_=1.8548 *m*_1_

*t*	−log *K*	*I*=0.010	0.015	0.020	0.025	0.030	0.035	0.040
						
		*m*_1_=0.002594	0 003891	0.005190	0.006485	0.007780	0.009080	0.01038
								
*°C*								
0	9.508	9.514	9.515	9.515	9.516	9.516	9.516	9.516
5	9.436	9.435	9.438	9.440	9.441	9.442	9.443	9.443
10	9.377	9.377	9.380	9.382	9.382	9.382	9.383	9.383
15	9.325	9.324	9.327	9.328	9.329	9.329	9.330	9.330
20	9.278	9.276	9.280	9.281	9.281	9.282	9.282	9.282
25	9.237	9.234	9.237	9.237	9.238	9.239	9.239	9.239
30	9.199	9.192	9.196	9.198	9.199	9.199	9.199	9.199
35	9.162	9.154	9.157	9.159	9.160	9.161	9.162	9.162
40	9.129	9.121	9.126	9.128	9.129	9.130	9.130	9.130
45	9.101	9.089	9.095	9.098	9.100	9.101	9.101	9.102
50	9.076	9.062	9.069	9.072	9.074	9.075	9.076	9.076
55	9.052	9.035	9.042	9.047	9.049	9.051	9.052	9.052
60	9.028	9.008	9.018	9.023	9.026	9.027	9.028	9.029

**Table 14:** p(*a*_*H*_γ_*Cl*_) in solutions composed of triethanolamine (*m*_1_) and hydrochloric acid (*m*_2_); *m*_2_=0.4860 *m*_1_

*t*	−log *K_bh_*	*I*=0.010	0.015	0.020	0.030	0.040	0.050	0.060	0.080	0.100
								
		*m*_1_=0.02058	0.03087	0.04116	0.06173	0.08231	0.10289	0.12347	0.16462	0.2058
										
*°C*										
0	8.291	8.407	8.430	8.449	8.475	8.493	8.510	8.525	8.552	8.577
5	8.173	8.291	8.315	8.335	8.361	8.378	8.394	8.409	8.437	8.462
10	8.067	8.186	8.209	8.227	8.253	8.272	8.288	8.303	8.330	8.358
15	7.963	8.081	8.105	8.125	8.152	8.168	8.183	8.198	8.226	8.249
20	7.861	7.979	8.002	8.022	8.050	8.067	8.082	8.096	8.123	8.146
25	7.762	7.883	7.907	7.926	7.951	7.968	7.984	7.998	8.026	8.048
30	7.666	7.786	7.809	7.827	7.853	7.870	7.885	7.899	7.927	7.952
35	7.571	7.692	7.715	7.732	7.758	7.775	7.790	7.805	7.832	7.856
40	7.477	7.601	7.623	7.641	7.664	7.681	7.699	7.714	7.739	7.761
45	7.387	7.511	7.534	7.551	7.575	7.593	7.607	7.622	7.649	7.673
50	7.299	7.423	7.446	7.463	7.488	7.506	7.521	7.536	7.562	7.584

**Table 15:** p(*a*_*H*_γ_*Cl*_) in solutions composed of tris (hydroxymethyl) aminomethane (*m*_1_) and hydrochloric acid (*m*_2_); *m*_2_=0.4961 *m*_1_

	−log *K_bh_*	*I*=0.01	0.02	0.03	0.04	0.05	0.06	0.07	0.08	0.09	0.10
									
		*m*_1_=0.02016	0.04032	0.06047	0.08063	0.10079	0.12094	0.14110	0.16026	0.18142	0.2016
											
*°C*											
0	8.850	8.946	8.981	9.004	9.021	9.035	9.049	9.061	9.071	9.081	9.090
5	8.677	8.777	8.809	8.834	8.851	8.864	8.877	8.890	8.901	8.911	8.922
10	8.516	8.614	8.649	8.673	8.690	8.704	8.718	8.730	8.741	8.752	8.762
15	8.362	8.461	8.493	8.518	8.537	8.552	8.566	8.578	8.588	8.598	8.607
20	8.214	8.315	8.345	8.370	8.390	8.405	8.419	8.431	8.441	8.451	8.460
25	8.075	8.176	8.207	8.232	8.251	8.266	8.280	8.292	8.302	8.312	8.321
30	7.934	8.037	8.069	8.095	8.114	8.129	8.142	8.153	8.164	8.175	8.186
35	7.803	7.907	7.936	7.961	7.982	7.998	8.012	8.023	8.035	8.046	8.056
40	7.677	7.781	7.811	7.836	7.857	7.872	7.885	7.896	7.908	7.920	7.931
45	7.554	7.660	7.691	7.715	7.735	7.750	7.764	7.776	7.788	7.800	7.811
50	7.437	7.543	7.574	7.599	7.618	7.633	7.647	7.660	7.672	7.684	7.694

**Table 16:** p(*a*_*H*_γ_*Cl*_) in solutions composed of 4-aminopyridine (*m*_1_) and hydrochloric acid (*m*_2_); *m*_2_=0.4962 *m*_1_

*t*	−log *K_bh_*	*I*=0.02	0.03	0.04	0.05	0.06	0.07	0.08	0.09	0.10
								
		*m*_1_=0.04031	0.06046	0.08061	0.10077	0.12092	0.14107	0.16122	0.18138	0.2015
										
*°C*										
0	9.873	9.992	10.016	10.037	10.056	10.072	10.085	10.096	10.107	10.118
5	9.704	9.825	9.850	9.872	9.890	9.906	9.919	9.931	9.942	9.953
10	9 549	9.668	9.694	9.716	9.735	9.750	9.763	9.774	9.785	9.796
15	9.398	9.519	9.543	9.565	9.582	9.597	9.610	9.623	9.634	9.645
20	9.252	9.375	9.399	9.419	9.437	9.453	9.466	9.477	9.488	9.499
25	9.114	9.236	9.259	9.279	9.297	9.313	9.326	9.338	9.348	9.358
30	8.978	9.103	9.125	9.145	9.162	9.177	9.190	9.202	9.212	9.223
35	8.845	8.970	8.993	9.013	9.031	9.046	9.058	9.070	9.081	9.091
40	8.717	8.842	8.864	8.885	8.904	8.920	8.933	8.945	8.955	8.964
45	8.594	8.717	8.741	8.763	8.783	8.799	8.813	8.824	8.834	8.844
50	8.477	8.603	8.624	8.646	8.666	8.682	8.694	8.705	8.716	8.727

**Table 17:** p(*a*_*H*_γ_*Cl*_) in solutions containing monoethanolamine (*m*_1_) and hydrochloric acid (*m*_2_); *m*_2_ = 0.5000 *m*_1_

*t*	−log *K_bh_*	*I*=0.010	0.015	0.020	0.025	0.030	0.040	0.050	0.060	0.070	0.080
									
		*m*_1_=0.02000	0.03000	0.04000	0.05000	0.06000	0.08000	0.10000	0.12000	0.14000	0.16000
											
*°C*											
0	10.306	10.390	10.412	10.429	10.443	10.456	10.476	10.492	10.506	10.519	10.531
5	10.133	10.219	10.241	10.258	10.272	10.283	10.301	10.318	10.333	10.345	10.357
10	9.965	10.054	10.074	10.091	10.105	10.117	10.137	10.153	10.167	10.179	10.191
15	9.803	9.892	9.911	9.928	9.943	9.955	9.976	9.992	10.005	10.017	10.029
20	9.647	9.735	9.756	9.775	9.790	9.802	9.821	9.836	9.850	9.861	9.872
25	9.500	9.590	9.612	9.629	9.643	9.654	9.673	9.690	9.704	9.717	9.729
30	9.351	9.441	9.461	9.480	9.495	9.507	9.527	9.544	9.558	9.570	9.580
35	9.209	9.300	9.320	9.338	9.353	9.366	9.387	9.404	9.418	9.429	9.439
40	9.071	9.163	9.182	9.199	9.215	9.229	9.251	9.268	9.282	9.294	9.303
45	8.939	9.033	9.052	9.067	9.082	9.096	9.119	9.138	9.151	9.162	9.173
50	8.811	8.903	8.920	8.937	8.953	8.969	8.993	9.010	9.023	9.034	9.045

**Table 18:** p(*a*_*H*_γ_*Cl*_) in solutions containing piperidine (*m*_1_) and hydrochloric acid (*m*_2_); *m*_2_ = 0.5003 *m*_1_

*t*	−log *K_bh_*	*I*=0.0265	0.0315	0.0365	0.0416	0.0516	0.0617	0.0717	0.0818	0.0918	0.1019
									
		*m*_1_=0.04997	0.05996	0.06995	0.07995	0.09993	0.11992	0.13991	0.15990	0.17988	0.19987
											
*°C*											
0	11.963	12.050	12.066	12.080	12.092	12.110	12.124	12.137	12.148	12.158	12.168
5	11.786	11.865	11.882	11.896	11.910	11.929	11.944	11.958	11.970	11.980	11.990
10	11.613	11.691	11.708	11.723	11.736	11.756	11.771	11.784	11.796	11.807	11.818
15	11.443	11.521	11.538	11.553	11.566	11.587	11.602	11.615	11.627	11.638	11.649
20	11.280	11.356	11.373	11.388	11.402	11.423	11.439	11.452	11.464	11.475	11.486
25	11.123	11.198	11.216	11.232	11.245	11.266	11.281	11.294	11.306	11.318	11.329
30	10.974	11.043	11.061	11.077	11.090	11.109	11.123	11.137	11.150	11.162	11.174
35	10.818	10.890	10.907	10.923	10.936	10.955	10.969	10.981	10.994	11.006	11.018
40	10.670	10.742	10.760	10.776	10.790	10.811	10.826	10.839	10.850	10.860	10.870
45	10.526	10.598	10.616	10.632	10.645	10.665	10.678	10.690	10.702	10.714	10.725
50	10.384	10.452	10.467	10.481	10.493	10.515	10.531	10.544	10.557	10.569	10.581

**Table 19:** p(*a*_*H*_γ_*Cl*_) in solutions of calcium hydroxide (*m*)

*t*	*m*=0.01500		0.01750		0.02000		0.0203 (Satd. at 25 °C)	
			
	*I*	*p*(*a*_H_*γ*_Cl_)	*I*	*p*(*a*_H_*γ*_Cl_)	*I*	*p*(*a*_H_*γ*_Cl_)	*I*	*p*(*a*_HCl_)
								
*°C*								
0	0.040	13.386	0.047	13.449	0.053	13.504	0.054	13.510
5	.039	13.161	.046	13.226	.052	13.285	.053	13.291
10	.039	12.958	.045	13.024	.051	13.082	.051	13.088
15	.038	12.769	.045	12.832	.051	12.887	.050	12.893
20	.038	12.584	.044	12.649	.050	12.706	.050	12.712
25	.037	12.414	.043	12 477	.049	12.531	.049	12.537
30	.037	12.250	.043	12.317	.049	12.375	.049	12.381
35	.037	12.095	.043	12.158	.048	12.213	.048	12.219
40	.036	11.954	.042	12.012	.048	12.064	.048	12.070
45	.036	11.809	.042	11.871	.047	11.920	.048	11.926
50	.036	11.674	.042	11.735	.047	11.786	.047	11.790
55	.035	11.545	.041	11.603	.046	11.654	.047	11.661
60	.035	11.418	.041	11.480	.046	11.534	.047	11.540

**Table 20:** p(*a*_*H*_γ_*Cl*_) in some solutions at 60 to 95 °C

Solution	t (°C)=60	70	80	90	95
					
0.05-*m* potassium tetroxalate	1.827	1.849	1.877	1.904	1.919
Potassium hydrogen tartrate, satd. at 25 °C	3.643	3.664	3.698	3.738	3.767
0.05-*m* potassium hydrogen phthalate	4.175	4.219	4.259	4.301	4.331
0.025-*m* potassium dihydrogen phosphate 0.025-*m* disodium hydrogen phosphate	6.948	6.962	6.979	7.001	7.014
0.01-*m* borax	9.026	8.990	8.953	8.920	8.899
